# Chinese Americans’ Use of Patient Portal Systems: Scoping Review

**DOI:** 10.2196/27924

**Published:** 2022-04-01

**Authors:** Katharine Lawrence, Stella Chong, Holly Krelle, Timothy Roberts, Lorna Thorpe, Chau Trinh-Shevrin, Stella Yi, Simona Kwon

**Affiliations:** 1 Healthcare Innovation Bridging Research, Informatics, and Design (HiBRID) Lab Department of Population Health New York University Grossman School of Medicine New York, NY United States; 2 Section for Health Equity Department of Population Health NYU Grossman School of Medicine New York, NY United States; 3 Division of Healthcare Delivery Services Department of Population Health NYU Grossman School of Medicine New York, NY United States; 4 NYU Health Sciences Library NYU Grossman School of Medicine New York, NY United States; 5 Division of Epidemiology Department of Population Health NYU Grossman School of Medicine New York, NY United States

**Keywords:** patient portal, electronic health records, personal health records, ehealth, health equity, digital divide, Chinese Americans, Asian Americans

## Abstract

**Background:**

Electronic patient portals are increasingly used in health care systems as communication and information-sharing tools and show promise in addressing health care access, quality, and outcomes. However, limited research exists on portal use patterns and practices among diverse patient populations, resulting in the lack of culturally and contextually tailored portal systems for these patients.

**Objective:**

This study aimed to summarize existing evidence on the access and use patterns, barriers, and facilitators of patient portals among Chinese Americans, who represent a growing patient population in the United States with unique health care and health technology needs.

**Methods:**

The authors conducted a literature search using the PRISMA Protocol for Scoping Reviews (Preferred Reporting Items for Systematic Reviews and Meta-Analyses-ScR) for extracting articles published in major databases (MEDLINE, Embase, and PsycINFO) on patient portals and Chinese Americans. Authors independently reviewed the papers during initial screening and full-text review. The studies were analyzed and coded for the study method type, sample population, and main outcomes of interest.

**Results:**

In total, 17 articles were selected for inclusion in the review. The included articles were heterogenous and varied in their study aims, methodologies, sample populations, and outcomes. Major findings identified from the articles include variable patterns of portal access and use among Chinese Americans compared to other racial or ethnic groups, with limited evidence on the specific barriers and facilitators for this group; a preference for cross-sectional quantitative tools such as patient surveys and electronic health record–based data over qualitative or other methodologies; and a pattern of aggregating Chinese American–related data into a larger Asian or Asian American designation.

**Conclusions:**

There is limited research evaluating the use patterns, experiences, and needs of Chinese Americans who access and use patient portal systems. Existing research is heterogeneous, largely cross-sectional, and does not disaggregate Chinese Americans from larger Asian demographics. Future research should be devoted to the specific portal use patterns, preferences, and needs of Chinese Americans to help ensure contextually appropriate and acceptable design and implementation of these digital health tools.

## Introduction

The expansion of health information technology (HIT) has provided patients with tools to proactively access their health information, self-manage chronic conditions, and communicate directly with providers [1]. In particular, electronic patient portals— which are secure internet-based platforms or websites that provide patients with 24-hour access to their personal health information—have emerged as a common communication and information-sharing tool for health care systems [2]. Patient portals offer a variety of features and functions for patients, such as the ability to access and review medical information, view lab and imaging results, schedule medical appointments and other visits, and interact with their health care providers [2-4]. Increasingly, these systems are directly integrated into electronic health record (EHR)–based platforms (eg, Epic MyChart or eClinical Works) or customer relationship management systems, as well as into the growing ecosystem of telehealth services. The COVID-19 pandemic expanded the use of patient portals as a facilitator of virtual health care and telemedicine, remote patient-provider communication, and monitoring [5-7]. Patient portals have demonstrated effectiveness in improving patient communication, engagement, and satisfaction [8,9], with some evidence on improvements in health outcomes [7,10] and lowered health care costs [6]. However, despite these benefits, adoption of and engagement with patient portals have varied, and significant disparities in the use of portal systems have been identified [2,11-18]. These disparities are shaped by individual, community, and structural factors such as social demographics (eg, socioeconomic status), health status (eg, disability diagnosis, chronic illness status), human-computer interface design challenges (eg, usability), and structural barriers (eg, lack of access to broadband internet).

Chinese Americans are a population frequently under- or mis-represented in health care, health delivery, and health research [19,20]. At roughly 5 million people, Chinese Americans comprise the largest subgroup of a heterogeneous community of Asian Americans and Pacific Islanders (AAPI), who themselves represent almost 10% of the US population [21-23]. Chinese American patients have distinct experiences interacting with the health care system [23,24], including care moderated by health technologies [25-27]. Although health disparities in this community have been identified and are mediated by factors such as language proficiency and immigration status [22,24], the details of these experiences are often obscured by problems with data collection and interpretation of health data that ignores the considerable heterogeneity and complexity of the AAPI designation [28].

To improve the effectiveness, acceptability, and use of digital health technologies such as patient portals among diverse communities, a better understanding of the use patterns and practices of the specific communities and their subgroups is needed. This scoping review summarizes the existing evidence on patient portal perceptions, adoption, and use among Chinese Americans, and it highlights gaps and areas for further research on patient portal and digital health technology use among Chinese Americans and other diverse patient populations.

## Methods

The aim of conducting a scoping review is to identify and broadly describe knowledge and research pertaining to a topic of interest as well as to identify trends, patterns, and gaps in the literature. Scoping reviews are ideal for research areas where the study question is broad or exploratory, there is limited literature on the topic, or study methodologies are diverse [29].

The review was conducted following the PRISMA Protocol for Scoping Reviews ((Preferred Reporting Items for Systematic Reviews and Meta-Analyses-ScR) [30]. In August 2020 and 2021, one of the coauthors (TR) who is an experienced medical librarian searched MEDLINE, Embase, and PsycINFO using the Ovid Platform and the Web of Science Core Collection. The search was not limited by language or publication date. Quantitative and qualitative studies that included primary data collection or data analysis were included; article types such as opinion pieces or letters to editors were excluded. The complete Ovid MEDLINE search strategy is available in Multimedia Appendix 1.

US-based studies that described the inclusion and perceptions of Asian Americans (eg, Asians, Asian Americans, Chinese Americans, and Filipinos) toward electronic patient portals were included. Studies that identified Asian Americans only under the heading of “Other” without additional specificity were excluded. Patient portals were defined as web-based platforms that provided access to data from EHRs, including features such as medical histories, visit summaries, medication lists, as well as secure messaging features, access to educational resources, and appointment scheduling [19,20,31]. Studies that focused primarily on the delivery of “real-time interactive” remote clinical care using audio or video communication technology (eg, synchronous telemedicine) [32] were excluded, as these technologies often exist separately from patient portal communication systems or do not support key asynchronous features such as personal health data review by patients or remote monitoring. Studies exploring general health information literacy or information-seeking via digital resources (eg, the internet) in this group were also excluded.

**Figure 1:**
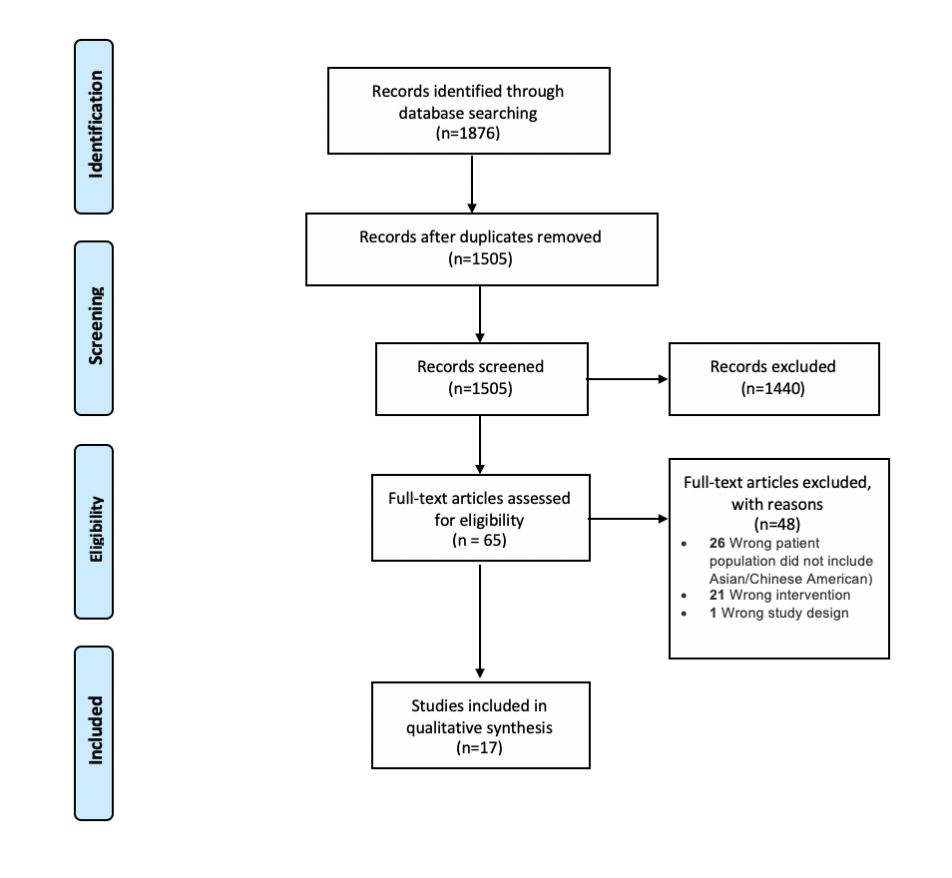
PRISMA Flowchart showing the screening and inclusion process of the studies. PRISMA: Preferred Reporting Items for Systematic Reviews and Meta-Analyses.

After duplicates were removed, 1505 articles remained. Titles and abstracts were screened using Covidence software [33] by 2 independent reviewers (SKC and HK) for explicit or implicit mention or identification of Chinese Americans. Conflicts were resolved through discussion between the 2 reviewers until consensus was reached. When needed, consultation was sought from another coauthor (KL) to reach consensus. The full texts, including tables, figures, and appendices, of 65 articles were reviewed following the same process. Ultimately, 17 articles were included in the analysis, as shown in Figure 1.

## Results

### Article Summaries

In total, 17 articles were selected for inclusion in the review. A summary of each article, including the study design, sample information (including the level of Asian American population identified in the study), and key findings can be found in Tables 1 and 2.

**Table 1:** Characteristics of studies that include identifiable data specific to Chinese Americans.

Study	Objective	Research design/tools	Sample population/level of Chinese American granularity and location	Patient portal technology/feature	Relevant results
Ackerman et al (2017) [33]	To understand the implementation of patient portals in safety net health care systems striving to meet MU^a^ criteria set by the Federal United States government	Mixed methods Rapid ethnography to assess MU, including interviews with providers and executives, informal focus groups with frontline staff, observations of patient portal sign-up procedures, and review of marketing materials and patient portal use. Administered modified version of the American Medical Association’s Health IT^b^ Readiness Survey Study tools: patient portal promotional flyers in English and Chinese at clinics; instructional video in Cantonese; language-congruent health staff available	5 California safety net health systems. Site 5 in Northern California, which serves *95% non-native English-speaking Chinese immigrants.* Location: Northern California	Patient portal (NextGen) implementation strategies and efforts at Site 5 Patient portal features: medical history, test results, secure messaging, and appointment requests	Overall: Community health centers were motivated by MU incentives to increase patient portal enrollment and integrate portal-related work into clinic routines. Barriers to patient portal usage for patients: lack of internet access, lack of computer proficiency, discomfort with portal use, language barriers, fear of government surveillance, and preference for in-person interaction with providers. Specific to Site 5: Chinese American patients face language barriers in accessing the patient portal. “The (EHR^c^ vendor) website isn’t in their Chinese language… How were they going to get their patients to be able to utilize this?” Perception that clinic discouraged staff from promoting patient portal once MU threshold was reached.
Gordon and Hornbrook (2016) [34]	To identify racial or ethnic and age disparities among older patients’ use of patient portals and access to digital technology and devices for email and web-based health care management programs	Quantitative (cross-sectional, administrative data and survey) Study 1: Analyzed administrative data about patient portal account status and use from the KPNC^d^ health plan Study 2: Mailed English survey questionnaire, from 2013 to 2014, to stratified random sample of Study 1’s population	Study 1: English-speaking Chinese (n=6314), non-Hispanic White (n=183,565), Black (n=16,898), Latino (n=12,409), and Filipino (n=11,896) older patients aged 65 to 79 years. Study 2: same as Study 1 Location: Northern California	KPNC internet-based patient portal, kp.org, and other digital health technology and tools (eg, emails, text, computer, smartphones)	Compared to Black, Filipino, and Latino older patients, Chinese and non-Hispanic White older patients were more likely to be registered to use the patient portal and more likely to use portal functions. Chinese and non-Hispanic White older patients were more likely to access digital devices, internet, and email. They were also more likely to be willing to use digital technology to seek health information.
Gordon and Hornbrook (2018) [35]	To assess disparities by race/ethnicity and age on older patients’ ability to engage with online health information and mobile health tools connected to their health system	Quantitative (cross-sectional, survey) Mailed English survey questionnaire, from November 2013 to February 2014 to members of the KPNC	Stratified random sample of 5420 *English-speaking* KPNC patients *Chinese (n=500)*, non-Hispanic White (n=1420), African American/Black (n=1500), Hispanic/Latino (1500), and Filipino (n=500) Location: Northern California	Digital health technology and tools (eg, internet, computer, mobile phone, email, text, social media, apps)	Chinese and non-Hispanic White older patients have higher levels of access to digital tools, experience in performing a variety of web-based tasks, and belief in their ability to seek health information on the internet compared to Black, Latino, and Filipino peers. Chinese older people prefer having telephone appointments with health coaches and are less interested in reading about health topics on the internet. Chinese older people have the lowest level of interest in using health apps.
Khoong et al (2020) [36]	To assess predictors of health technology use (eg, language preferences, smartphone ownership, type of clinic for health care)	Quantitative (cross-sectional, survey)	Nonrandom sample of 1027 participants *Chinese-speaking Chinese (n=257)*; Spanish-speaking Latino (n=256); English-speaking non-Hispanic Black (n=514); English-speaking non-Hispanic White (n=43); and English-speaking Latino (115) Location: San Francisco, California	Digital health technology and tools for communication with clinicians (eg, email, text, phone apps, web-based health videos, and online health support groups)	Relative to English-speaking survey respondents, individuals who preferred the Chinese language had lower odds of texting or using an app to communicate with their clinician. There were no differences in using emails or watching web-based health videos. Language concordance was suggested as a major barrier.

^a^MU: meaningful use.

^b^IT: information technology.

^c^EHR: electronic health record.

^d^KPNC: Kaiser Permanente Northern California

**Table 2:** Characteristics of studies with aggregated Asian American data.

Study	Objective	Research design	Sample population/ level of Chinese American granularity and location	Focus	Relevant results
Ahlers-Schmidt and Nguyen (2013) [37]	To obtain parents’ feedback and intention to use patient portals for their children’s health records and concerns post the facilitated learning session	Quantitative (cross-sectional, survey)	Parents of patients. (N=65) White (n=26, 40%); Hispanic (n=14, 22%); *Asian (n=9, 14%)***;** African American (n=6, 9%); Mixed/other race (n=8, 12%) Location: Kansas	University of Kansas Pediatric Clinic’s eClinical Works, an electronic medical record with a patient portal	Most parents did not know about the patient portal before the study demonstration. Parents expressed that patient portal was simple to use after demonstration. Parents liked portal functions such as viewing lab results and medical records; disliked need to make separate accounts for each child and the lack of a symptom checker function.
Dalrymple et al (2018) [38]	To assess parents’ use of the internet for health information and parents’ awareness of digital health technologies to obtain health information Screening questions assess parents’ level of health literacy and interest in use of patient portals	Quantitative (cross-sectional, survey) Study tool: 26-question paper and pencil survey adapted from interview protocol designed from previous study	Total sample population of parents or adult caregivers of children and adolescents, N=270 *Asian (1.9%)*; American Indian/Alaska Native (1.5%); Black/African American (38.1%); Hispanic/Latino (13.7%); Native Hawaiian/Pacific Islander (0.4%); White (40.7%); more than one race/ethnicity (4.4%); and Other (1.5%) Location: Unspecified large metropolitan area in eastern United States	Internet and patient portal	Most patients reported having access to the internet and using the internet to seek general and health information. Respondents expressed enthusiasm and interest in using a patient portal if it were available from their health care provider.
Foster and Krasowski (2019) [39]	To assess patient portal usage by ED^a^ patients at an academic medical center using patient portal activation rates and rates of accessing diagnostic test results on patient portals	Quantitative (retrospective cohort, EHR^b^, and administrative data)	25,361 unique ED patients identified via EHR patient portal records *Asian (n=451)*; African American/Black (n=2,254); White (n=20,637); Hispanic/Latino (n=1257); Other (n=762) Location: Iowa	UIHC^c^ patient portal (MyChart), connected to EPIC EHR system	Highest rates of using the patient portal to view laboratory and radiology results were observed for younger female, proxies, Asian, and White patients. Activation rates were highest for Asian and White patients. Disparities were observed among teenagers, older adults, African American/Black, and Hispanic/Latino patients.
Goel et al (2011) [12]	To examine the enrollment in and use of patient portal at an academic medical center by race/ethnicity, gender, and age	Quantitative (cross-sectional, EHR and administrative data) Study tool: patients’ use of EHR-based advice function and request for refills	Patients enrolled in the patient portal system, N=7088 *Asian (n=142, 2%)*; White (n=3472, 49%); Black (n=1063, 15%); Latino (n=284, 4%); Other (n=851, 12%); Missing race/ethnicity (n=1347, 19%) Location: Chicago	Northwestern Medical Faculty Foundation’s EHR patient portal	Significant disparities in patient portal enrollment by race/ethnicity were observed, but not by age or gender. White patients (74%) were more likely to enroll in patient portals compared to Black (55%), Latino (64%), and Asian (66%) patients. When adjusted for variables (eg, age, gender, income, education, and provider effects), the disparity between Asian and White patients was no longer statistically significant.
Graetz et al (2016) [40]	To assess sociodemograp hic disparities in patient portal use	Quantitative (cross-sectional, survey) Study tool: Administered paper-based survey mailed to participants; survey measures internet access, secure email use, care preference, sociodemographics, and health characteristics	Total study participants from KPNC^d^, N=1041 White (n=617, 59.3%); *Asian (n=145, 13.9%)*; Black (n=122, 11.7%), and Hispanic (n=12812.3%) Location: Northern California	Internet and email	Asian and Black respondents were more likely to rarely or never to use the internet (45.4% and 45.6%, respectively) compared to their White respondents. Asian participants (78%) preferred in-person care over telephone care compared to White patients (64%).
Ketterer et al (2013) [41]	To identify predictors of patient portal enrollment and activation among a pediatric primary care population	Quantitative (cross-sectional, EHR and administrative data) Study tool: primary care database, and enrollment in and use of a patient portal	Total sample population N=84,015 Black (n=35,286, 42%); *Asian (n=2520, 3%)*; White (n=35,286, 42%); Hispanic (n=10,082, 12%); Other (n=9242, 11%); and Unknown (n=1680, 2%). Location: Delaware	Patient portal site, MyNemours	Adjusted odds of portal enrollment were lower for Asian respondents compared to White respondents. Once enrolled, there was no difference in portal activation between Asian respondents and White respondents. Study suggested language concordance as a major barrier.
Lyles et al (2013) [42]	To understand how patient-provider relationships influence patients’ use of online patient portals and secure messaging	Quantitative (cross-sectional, survey)	Surveyed patients DISTANCE^e^ Black (23%); Latino (16%); *East Asian (ie, Chinese, Japanese, Korean, or Vietnamese) (10%)*; Filipino 12%); and Other (6%) Location: Northern California	KPNC’s internet-based patient portal, kp.org	White and Latino individuals with higher trust in the providers were more likely to register on the patient portal. There was no relationship between trust in provider and patient portal use for Asian respondents.
Lyles et al (2016) [43]	To determine whether racial/ethnic minority patients’ use of the patient portal’s medication refill function has changed over time compared to White patients	Quantitative (EHR and administrative data) Study tool: diabetic patients’ use of EHR-based medication refill function	White (58%); *Asian (10%)*; Latino (9%); Filipino (9%); Black (7%); and Mixed/other (9%) Location: Northern California	KPNC’s internet-based patient portal, kp.org	Asian were not less likely to exclusively use refill functions than other ethnic groups. Adherence to medication refills improved over time for all ethnic groups, but there was no significant difference between ethnicities. Usability and accessibility were identified as barriers to portal registration.
Miles et al (2016) [44]	To measure and evaluate the frequency at which patients use the patient portal to view online radiology reports	Quantitative (cross-sectional, EHR and administrative data) Study tool: patient interactions with portal features (eg, radiology, laboratory, and clinical notes) and sociodemographic factors	*Asian or Pacific Islander (n=6376, 10.4%);* American Indian or Alaska Native (n=522, 0.8%); Black or African American (n=3817, 6.2%); Hispanic or Latino (n=1850, 3%); White (n=44,163, 72.25); and Other/more than one race (n=675, 1.1%); and Unknown (n=3728, 6.1%) Location: Seattle, Washington	UW’s^f^ patient portal system, UW eCare web portal	Asian respondents were more likely than White patients to view their radiology reports. Older patients, primary non-English speakers, and those with non-commercial insurance viewed reports at lower rates. Concerns identified in the study include loss of patient confidentiality, health information inaccuracy, and disruption of patient-physician relationship.
Patel et al (2011) [45]	To determine low-income, ethnically diverse consumers’ attitudes and beliefs toward HIE^g^ and use of HIE via PHRs^h^ and to identify factors that impact consumers’ support for providers’ use of HIE and their own personal use of PHRs	Quantitative (cross-sectional, survey) Study tool: survey adapted from previously validated national surveys. Survey was translated into Spanish, Russian, and Mandarin Chinese	BHIX^i^’s patients White (n=36, 74%); *Asian (n=57, 28%)*; African American (n=20; 10%); and Other (n=56, 27%). *Spoke Chinese at home (n=42, 20%)* Location: New York City, New York	EHRs, internet, HIE, and PHRs	Compared to other racial/ethnic groups in the study, Asian Americans indicated lower levels of support for HIE (48%) and lower levels of potential PHR usage (67%).
Sarkar et al (2010) [46]	To examine whether use of an internet-based patient portal differed between English-speaking patients with limited health literacy and English-speaking patients with adequate health literacy	Quantitative (cross-sectional, survey) Study tool: DISTANCE study was conducted in English, Spanish, Cantonese, Mandarin, and Tagalog	Total of 14,201 surveyed participants from DISTANCE study Non-Hispanic White (n=3957, 28%); Latino (n=1923, 14%); African American (n=2899, 21%); *Asian (n=1253, 9%)*; Filipino (n=1624, 12%); Other (n=2446, 17%) Location: Northern California	KPNC’s internet-based patient portal, kp.org	Study did not find increased risk of not signing onto the patient portal for Asian Americans compared to African American, Latino, and Filipino respondents. Asian Americans had lower rates of never using patient portal functions including lab result viewing, medication refills, email, and scheduling appointments. Health literacy was identified as a barrier to portal activity.
Sarkar et al (2011) [47]	To examine portal use habits via the frequency at which participants requested a password for the patient portal, the proportion of participants who activated their accounts by changing the default password, and the proportion of participants who login to their accounts using their personal, customized password	Quantitative (cross-sectional, EHR and administrative data ) DISTANCE study was conducted in English, Spanish, Cantonese, Mandarin, and Tagalog	Total of 14,201 surveyed participants from DISTANCE Study Non-Hispanic White (n=3957, 28%); Latino (n=1923, 14%); African American (n=2899, 21%); *Asian (n=1253, 9%)*; Filipino (n=1624, 12%); Other (n=2446, 17%) Location: Northern California	KPNC’s internet-based patient portal, kp.org	Asian American (53%) and White (51%) participants were more likely than their African American (31%), Latino (34%), and Filipino (32) counterparts to request a password for the internet-based patient portal and to login to the patient portal after requesting a password. Older adults with less educational attainment were less likely to register and use the patient portal.
Tieu et al (2017) [48]	To measure participants’ satisfaction with use of patient portal	Mixed methods (cross-sectional, usability testing and survey) Study tool: Conducted English language performance testing and think-aloud interviews with participants and administered survey to participants	Total of 25 English-speaking (23 patients and 2 caregivers) participants. African American (n=9, 36%); White (n=6, 24%); Hispanic (n=2, 8%); *Asian or Pacific Islander (n=5, 20%)*; and Other (n=3, 12%) Location: San Francisco, California	RFPC’s^j^ patient portal, MYSFHEALTH	Participants with limited health literacy, including Asian and Pacific Islander patients were more likely to need assistance navigating the patient portal. Barriers to patient portal use for participants with limited health literacy include (1) lack of basic computer skills; (2) routine computer use challenges despite basic knowledge of computers; (3) difficulty reading, writing, and understanding language; and (4) difficulty understanding and applying medical information from the internet and patient portal.

^a^ED: emergency department.

^b^EHR: electronic health record.

^c^UIHC: University of Iowa Hospitals and Clinics.

^d^KPNC: Kaiser Permanente Northern California.

^e^DISTANCE: Diabetes Study of North California.

^f^UW: University of Washington.

^g^HIE: health information exchange.

^h^PHRs: personal health records.

^i^BHIX: Brooklyn Health Information Exchange.

^j^RFPC: Richard H. Fine People’s Clinic.

The included articles varied in terms of the study methodology, sample population, data collection methodology, and geographic area within the United States. Among these, 10 were from populations in California [34-36,40,42,43,45-48], with 5 from the Kaiser Permanente health system [35,36,42,43,46]. Further, 3 studies used a shared database—the Diabetes Study of Northern California (DISTANCE)—to analyze portal-related outcomes [40,47,48]. Of the data collection tools described in these studies, 5 studies indicated they were available and conducted in Chinese (eg, Mandarin or Cantonese) [12,34,43,47,48].

Overall, the articles described heterogenous results among varied patient populations, health conditions, and care settings. Few clear themes emerged and results specific to Asian American subgroups such as Chinese Americans were not identified. In general, the authors were able to identify the following major themes and trends from the results.

1. Chinese Americans demonstrate variable patterns of patient portal access and use as compared to other demographics, particularly racial or ethnic groups; exploration of the specific contexts of use, including barriers and facilitators, is limited.
2. Most studies employed cross-sectional, quantitative tools to assess patient portal use patterns and practices, including patient surveys and EHR-based data that measure portal activity (eg, logins and click-throughs); neither longitudinal nor significant qualitative research studies were conducted to validate or further explore nuances in findings specific to Chinese Americans.
3. Despite the heterogeneity of the populations included in AAPI designation, studies exploring patient portals do not disaggregate Asian and Asian American study populations into Chinese Americans and other subgroups.

### Findings Specific to Chinese Americans

Only 4 studies [33-36] specifically disaggregated Chinese American populations (Table 1). All 4 of these were from California. Among these, 3 [34-36] were primarily based around surveys, and 1 [33] was based on rapid ethnography, mostly focusing on understanding the barriers to accessing patient portals. Barriers reported included language barriers, lack of internet access or computer proficiency, fear of government surveillance, and a preference for in-person interaction. Further, 2 of the studies [34,35] found that Chinese patients were more likely than other non-White groups to register and use internet-based portals, and 1 [40] found that relative to English-speaking respondents, people who preferred the Chinese language were less likely to send text messages or use an app to contact their clinician.

#### Chinese Americans Demonstrate Variable Patterns of Patient Portal Access and Use Compared to Other Racial or Ethnic Groups

This represents a finding in the data across studies, with some demonstrating lower rates of use and others demonstrating higher rates and rates comparable to White patients. In a study on the use of the Northwestern Medical Faculty Foundation’s electronic patient portal [44], the authors found that once variables such as age, gender, education, income, and provider effects were adjusted, there was no disparity between the enrollments of Asian American and White patients on the patient portal. In another study of Chinese American older adults in Kaiser Permanente, Northern California [35], the authors found that non-Hispanic White and Chinese American older adults were more likely than other racial or ethnic groups to register for using the portal and its functions such as sending messages, viewing lab results, or ordering prescription refills. Other studies showed lower use and lower motivation to use digital health technology among Chinese Americans. In their study examining patients’ patterns of texting and communication with their clinicians via apps, Khoong et al [36] found that individuals who preferred to use Chinese language had lower odds of texting or using an app to communicate with their clinicians compared to English-speaking survey respondents. In a study assessing older patients’ readiness to use eHealth tools, researchers found that Chinese American patients had the lowest level of interest in using patient portal technology among all the racial or ethnic groups in the study, though their experience of using the internet was similar to that of non-Hispanic White patients [36]. In their assessment of attitudes toward health information exchanges (HIEs) and personal health records (PHRs), Patel et al [45] found that Asian Americans were less likely than other racial or ethnic groups to support the use of PHR technology.

Identified studies provided limited evidence on the barriers faced by Chinese Americans in using patient portals. For individuals, the main reported barrier was language congruency with the portal or related technologies, or English language proficiency. In a mixed methods study evaluating the implementation of meaningful use at community health centers in California, Ackerman et al [33] noted that many patients could not read English and that even if communication with care providers could be conducted in Chinese, most EHR features (including records, test results, and communication tools like the patient portal) were exclusively in English. The authors also noted concerns among some Chinese Americans about government surveillance, particularly among patients who were undocumented or had concerns regarding their immigration status. Additional individual-level barriers identified in the studies included issues of usability and accessibility of the portal tool [43], concerns around confidentiality and privacy [38], low health literacy, [48], and digital literacy [45]. Conversely, in a study assessing the influence of patient-provider relationships on patient portal and messaging usage, Lyles et al [42] found that although trust in providers was correlated with registration for portals by White and Latinx patients, this was not the case for Asian patients.

Identified community and structural barriers were largely related to clinic-level resources and included the clinical staff’s ability to support patients’ engagement in patient portal technology and the paucity of language-congruent support services. In their rapid ethnography with clinical staff in safety net hospital–affiliated practices, Ackerman et al [33] reported challenges related to providers and staff members having limited time and skills to coach patients in using the patient portal, and concerns regarding meaningful use metrics that prioritize outcomes such as portal sign-up rather than sustained use. The researchers also identified disruptions to clinical workflows and increased administrative burden as barriers to effective implementation and use of EHR-related tools. In 3 studies, access to digital technology and infrastructure such as the internet was associated with higher rates of patient portal access and use by Chinese and Asian American patients [35-37].

#### Most Studies Employed Cross-sectional Quantitative Tools to Assess Patient Portal Use Patterns and Practices, Including Patient Surveys and EHR-Based Data That Measure Portal Activity

Among the 17 studies, 8 employed survey-based, numeric (eg, Likert scale) data collection tools disseminated using either digital tools (eg, email) or in person. Survey question areas ranged from portal familiarity and general perspectives to personal experiences, feature preferences, and self-reporting of details on use habits [12,35-37,39,40,43,47]. The remaining studies used either administrative information–based EHRs or associated databases. Furthermore, 6 studies conducted primary EHR-based analyses to identify patterns and trends in portal-based activities [38,41,44,46,48,49]. Key EHR- and portal-based measures reported by researchers included patient portal registrations [35,44], logins and appointment booking [47-49], medication refill requests [46], viewing of results and reports (eg, radiology reports) [38,41,47], and texting and other forms of communication with clinicians [40,42]. These activities were analyzed for frequency and other patterns, and they were often compared among demographics such as age, race or ethnicity, sex or gender, income level, insurance status, and language. Key themes in the survey questions included actual and expected use of different features, concerns and barriers related to using portals, and confidence in the ability to use portals and understand health information shared through these portals. Most of these measures are applied cross-sectionally, and there is neither longitudinal nor significant qualitative research to validate or further explore nuances in findings specific to Chinese Americans or other Asian American subgroups. No studies included measures of associated health outcomes.

#### Despite the Heterogeneity of the Populations Included in AAPI Designation, Studies Exploring Patient Portals Largely do not Disaggregate Asian and Asian American Study Populations

Of the 17 studies included in this review, only 4 specifically disaggregate or discuss Chinese Americans [34-36,40]. The remaining studies generally refer to “Asian Americans” or “Asians,” with only indirect references to over 20 unique ethnic subgroups included in that designation or otherwise included in the study sample, data collection, or analysis. For example, Chinese-speaking patients were occasionally mentioned in the text or tables of these studies [12,34,43,47,48] but not included in any multivariate analyses as a separate category. In these studies, it was inferred that Chinese American patients were included via references to the languages of the data collection instruments (eg, Mandarin or Cantonese) or the study database being used for analysis. No studies specifically or exclusively evaluated Chinese Americans’ attitudes toward, perceptions about, or use of patient portal technology.

## Discussion

### Principal Findings

This scoping review highlights the extremely limited research on the use patterns, experiences, and needs of Chinese Americans who access and use patient portal systems for their health care. The identified studies were heterogenous in their approaches and outcomes, making generalizable trends in the data difficult to identify, although we were able to identify some patterns in the research methodologies and data collection tools across studies. By and large, the existing studies have focused on the identification of varying portal use patterns among racial, ethnic, and other demographics, and their correlative predictors such as age, primary language, or health literacy. Overall, the studies obtained mixed findings regarding the rates of portal usage by Chinese Americans when compared to other populations, with some indicating lower rates of portal adoption and use when compared to White patients and others finding comparable rates. We were unable to identify trends more granularly in terms of portal access within Chinese American subgroups (eg, women, geographic populations) due to limitations in the available data. We identified individual- and system-level factors that contributed to use patterns, as well as barriers to access and usage. Relevant individual-level factors included English language proficiency and language congruency with portal technology; health literacy; perceived usability and usefulness of the technology; and trust in provider relationships, privacy, and confidentiality. Relevant system-level factors included clinical resource and capacity limitations, and access to digital tools such as email and the internet. Studies tended to be cross-sectional and quantitative in nature, with minimal exploration of longitudinal trends in use patterns or practices, qualitative aspects, or correlation with health outcomes. Finally, we identified a pattern of data aggregation practices that tended to combine and compare Asian Americans as a larger demographic group to other racial or ethnic groups, rather than identifying data at the level of Chinese Americans or other subgroups. This practice had the effect of generalizing learning across Asian Americans, thus providing limited insight into the experiences of Asian subgroups of different ethnicities, languages, and religious affiliations, among other factors.

To our knowledge, this is the first study to evaluate the patient portal use patterns and needs of Chinese Americans. Prior research has explored various features of patient portal activity, use, and experience in other clinical contexts, including among Black and Latinx communities and vulnerable populations such as the older people and those with disabilities [50-52]. A comprehensive review of interventions to increase patient portal use in “vulnerable populations” by Grossman et al in 2019 [4] identified 18 studies evaluating the impact of interventions designed to increase portal use or reduce disparities in use. The authors noted that most studies focused on individual-level interventions such as patient education and training and identified a lack of interventions or programs targeting tool- (eg, patient portal interfaces or features), community-, organizational-, or system-level factors to improve portal adoption and use [4]. This is also supported by the findings of the study led by Antonio et al [52] that explored patient portal research through the lens of health equity and identified a varying and often superficial level of interest in portal technology among underserved groups by researchers and an underemphasis on the systemic factors influencing patient portal access and use among diverse communities. Although comprehensive, these reviews included limited information on the needs, use patterns, or potential interventions for specific vulnerable groups, particularly among racial or ethnic demographics; as observed in our findings, data on race and ethnicity included in these reviews often excluded Asian Americans or did not identify Asian American subgroups. Though our study includes some of the articles referenced by these reviews, our focus on Chinese and Asian American subgroups provides additional specificity to the overall literature on patient portals and exposes existing challenges in identifying and applying appropriately tailored solutions to technical problems for undifferentiated “vulnerable” patients.

The findings of this study have important implications for the design and deployment of patient portals and other digital health tools (eg, EHRs, mobile health apps) as well as for the study of health technology usage among Chinese Americans, Asian subgroups, and other diverse or vulnerable patient populations. Overall, there is need for a more granular study focusing on the use of digital health technology by diverse communities to elucidate key differences in their needs, preferences, and constraints. Participatory design frameworks that incorporate diverse stakeholders to identify and address specific needs, preferences, and concerns regarding health care technologies can help inform more effective and sustainable implementation of these tools in clinical practice. Frameworks and methodologies that explicitly address digital health disparities and digital health equity, such as the equity-centered design framework [53] and the digital health equity framework [54], can additionally help identify and overcome structural barriers such as access to digital infrastructure or institutional racism. At the same time, there is a need for clearer definitions and more granular breakdowns of populations included in data collection and data publication processes to better inform appropriate, targeted recommendations for diverse communities. Critically, the use of aggregate data as a proxy for subsets of Asian American patients obscures differences in patient- and community-level experiences or needs and conflates the experiences of minority communities within that population. This problematic practice has been well documented, and efforts are in place to address it in research and clinical practice [27,55-57]. Health informaticists and technology researchers can be change leaders in this area by applying well-established design practices such as user stories, personas, and customer segmentation to clearly identify the needs of patient users, including those that are defined by a specific cultural identity or intersections of identities [58,59].

There are several limitations to this study. We included only major databases (PubMed and Embase) and did not include unpublished or gray literature. We also limited our inclusion criteria to articles published only in English, excluding Chinese language biomedical databases such as the China National Knowledge Infrastructure. We further included only those articles focusing on populations in the United States. These criteria were established to ensure a focused review of our target community of interest, namely Chinese Americans, engaging with relatively similar health care delivery models and HIT technology. However, this may have resulted in the exclusion of relevant articles, particularly those published in Chinese language journals. Additionally, although the term “patient portal” included in our search string is broadly used, our search may have missed studies that incorporated portals, portal-like systems (eg, PHRs), or portal features without explicitly identifying them. We attempted to address this by performing a series of web-based searches (Google) and manual searches to identify articles using variable terms that could meet our inclusion criteria. Finally, our study did not systematically evaluate the quality of the data presented in the included studies beyond an assessment of the study design and the level of racial or ethnic granularity among Asian Americans; moreover, we did not evaluate the bias in these studies. Future areas of research may include expanded language contexts and further quality and bias evaluations.

### Conclusions

There is limited research dedicated to understanding the use patterns, experiences, and needs of Chinese Americans who access and use patient portal systems for their health care. Most of the research in this area focuses on disparities in use and access across the aggregated racial and ethnic demographic of Asian Americans, potentially obscuring important differences among and between the diverse and heterogeneous populations that comprise this designation. Studies are also overwhelmingly quantitative, focused on surveys and administrative data from portal systems, and they lack longitudinal data. Future research should focus specifically on Chinese Americans and prioritize performing more detailed longitudinal and qualitative evaluations to understand why specific communities of patients access and use portals in the ways that they do. A broader understanding of the diversity of health technology users in general can help ensure that these tools are applicable and acceptable to all patients, including the most vulnerable, and do not contribute to disparities in health access, equity, or outcomes.

## Appendix

Multimedia Appendix 1 Ovid MEDLINE search strategy.

## Abbreviations

AAPI: Asian Americans and Pacific Islanders
DISTANCE: Diabetes Study of Northern California
EHR: electronic health record
HIE: health information exchange
HIT: health information technology
PHR: personal health record
