# Supplementary figures and images for: Chinese Americans’ Use of Patient Portal Systems: Scoping Review
Source: JMIR Hum Factors. 2022 Apr 1;9(2):e27924. doi: 10.2196/27924 (PMC9015766; doi:10.2196/27924)

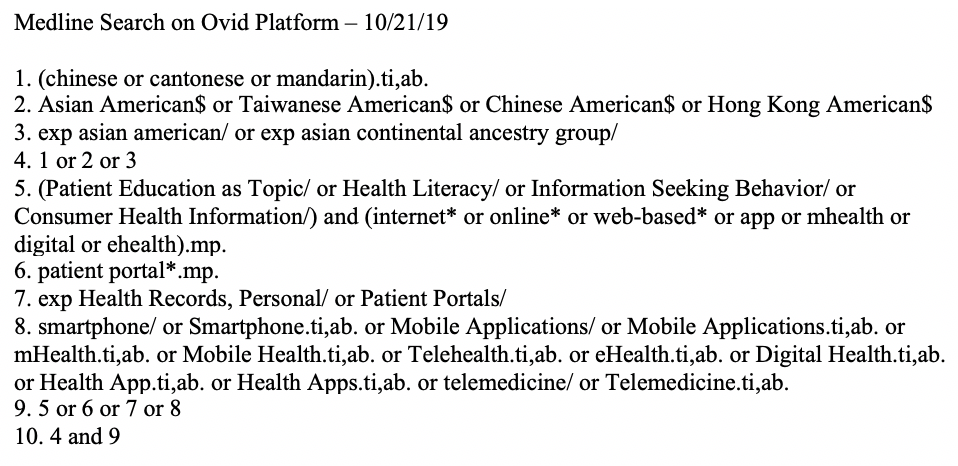

Supplement: Multimedia Appendix 1 [file humanfactors_v9i2e27924_app1.png]
